# Simulation Enhances Soft Skills Among Inter-Professionals Participating in an International Service-Learning Experience to a Resource-Limited Country

**DOI:** 10.15694/mep.2019.000024.1

**Published:** 2019-02-05

**Authors:** Suzanne Trotter, Sharon Dunnivan-Mitchell

**Affiliations:** 1 University of St. Augustine for Health Sciences

**Keywords:** Inter-professional education, simulation, international service-learning, physical therapy, occupational therapy

## Abstract

This article was migrated. The article was marked as recommended.

**Introduction:**Soft skills are the hallmark of a master healthcare provider. One of the most effective ways to teach soft skills to healthcare providers is through service-learning. Evidence suggests that adding simulation as part of a service-learning team’s pre-departure training (PDT) will better prepare them to resolve ethical and cultural dilemmas often encountered in resource-limited countries, plus facilitate soft skills. We hypothesized that simulation could improve soft skills of physical and occupational therapy students and clinicians providing rehabilitation services on a one-week service-learning experience in Guatemala.

**Methods:**A convenience sample of 21 physical and occupational therapy students and clinicians who participated in four 1-hour PDTs were included in this qualitative study using grounded-theory methods. Training consisted of didactic, reflective and simulation components designed to introduce self-awareness, team-building, cultural knowledge, and to support trip preparations. Four debriefings were recorded using open-ended questions with a thematic approach around the concepts of preparedness and cultural adaptability, which represented the dependent variables. The independent variable was a 20-minute simulation emphasizing cultural and socio-emotional challenges of the host community.

**Results:**Six themes emerged: confidence, empathy, communication, mentorship, self-knowledge, and cultural competency. The themes described were core elements of empowering the participants towards advocacy and process improvement. As a result of the simulation experience, participants in this study were better able to respond to distressing situations encountered on site, and they expressed the service-learning experience, supported by the PDT simulation, stirred significant maturation.

**Discussion and Conclusion:**Simulation is useful for developing self-regulatory skills, especially in response to culturally novel, emotionally-charged situations. Simulation for enriching international service-learning experiences is recommended as best practice to prepare healthcare providers in facing ethical and cultural demands of resource-limited countries.

## Introduction

Soft skills, which encompass many elements of emotional intelligence, combined with clinical competency, are the hallmark of a master healthcare provider and flow effortlessly within a patient interaction. Emotional intelligence, compassion, communication, empathy, leadership, self-awareness, cultural competency, confidence, self-regulation and more, have been identified as essential elements which make up the soft or non-technical skills of a clinician delivering quality, patient-centered care ( Liebrecht & Montenery, 2016). Educational institutions increasingly seek to incorporate curricular content emphasizing the development of these elusive soft skills in their students. One of the most effective ways to teach these skills to healthcare providers is through service-learning ( Moran et al., 2015; Pechak, Gonzalez, Summers & Capshaw, 2013). To reduce the stress experienced by providers while serving in under-resourced environments, as well as ensure mutual benefit for the host community, adequate pre-departure training (PDT) is essential.

A single exposure to crisis using simulation can significantly improve the nontechnical skills of anesthesia residents ( Yee et al., 2005).Evidence suggests that adding simulation to PDT will better prepare providers to resolve ethical and cultural dilemmas ( Ekelman, Bello-Haas, Bazyk, & Bazyk, 2003; Mu et al., 2016) while facilitating soft skills ( Johnson & Howell, 2017; Buff et al., 2015). However, it has not yet been utilized for an interprofessional PDT of rehabilitation providers. We hypothesized that the use of simulation for pre-departure training could improve the soft skills of physical and occupational therapy students and clinicians providing rehabilitation services on a one-week service-learning experience in Guatemala.

## Methods

*Simulation Curriculum Development:* A 20-minute simulation scenario was developed for one pre-departure training. It centered around a case requiring two English-speaking student therapists to teach a Spanish-speaking caregiver of a wheelchair dependent child how to perform stretches to improve mobility and quality of life. The goal of the simulation was to teach appropriate communication utilizing a translator and to allow students to exhibit empathy in a culturally-stressful situation. The topic was selected based on previous interactions with patients from earlier trips to this destination. Table 1 lists the simulation case and learning objectives, that are based on global health competencies for health providers ( Jogerst et al., 2015). A 30-minute debriefing followed the simulated experience.

**Table 1.** Clinical case and learning objectives for simulation scenario emphasizing communication skills utilizing a translator

Case: 14-year-old nonverbal wheelchair dependent female is brought to the rehabilitation outpatient clinic by her Spanish speaking mother who wants to learn ways to better care for her daughter at home. Within the patient interaction it becomes apparent that the mother experiences distress due to lack of support at home because her husband drinks all day and her son was killed recently due to gang violence. Interprofessional English-speaking students will interact with the patient and mother via a translator.
Learning Objectives: • Prepare healthcare providers for the emotional challenges of working in a resource-limited setting by exhibiting caring and compassion when interacting with the patient and family member. • Prepare providers to communicate and collaborate effectively with other disciplines to optimize patient outcomes. • Prepare providers to communicate safe instructions and care for patient and family members via a translator.

*Participants:* A convenience sample of 21 physical and occupational therapy students and clinicians who participated in four 1-hour PDT sessions before traveling to Guatemala were included in this qualitative study using grounded-theory methods. Informed consent was obtained from all participants once accepted to the service-learning project. They were made aware that participation in the study was independent of the project, there was no obligation to participate, and they could withdraw at any time. Pre-departure training consisted of didactic, reflective and simulation components designed to introduce self-awareness, team-building, cultural knowledge, and trip preparations. All training occurred face to face and via Skype for team members who were not physically able to attend. To be included in the study, all participants had to attend 100% of the training. Twenty-five participants began in the study, but three were excluded for not attending all the training. Ethics approval was granted by the Institutional Review Board of The University of St. Augustine for Health Sciences (UR-0809-228). Written and verbal informed consent was obtained from all participants before each stage of data collection.

*Debriefings:* Four total debriefings were recorded using open-ended questions with a thematic approach. The first briefing occurred before the PDT. The second occurred after the simulation. The last two debriefings occurred at the first and final day in Guatemala. Debriefing topics addressed cultural preparedness, adaptability, inter-professional collaboration, problem-solving, teamwork, and impact on professional development. These themes are considered a priori in that they are current topics that have been identified in association with global health education (Josgerst et al., 2015; Logar, Le, Harrison, & Glass, 2015; Ventres & Wilson, 2015). Participants had the opportunity to share what they learned, ask questions, and express their feelings regarding their experience working in the Guatemalan community, and specifically how simulation prepared them for any experiences that were encountered during the week. Participants excluded from the study were asked to refrain from the recorded discussion. However, they were encouraged to share after the study participants were recorded, to not invalidate the debriefings. The dependent variable was subject preparedness and adaptability. The independent variable was a 20-minute simulation emphasizing cultural and socio-emotional challenges of the host community.

*Data Analysis:* Transcription of the recorded debrief sessions were executed verbatim without subject identification. Transcript review and thematic coding were performed independently by both researchers followed by several meetings at various intervals. A priori coding using the thematic areas identified previously were observed; however, the researchers were also aware of emerging themes. Two-member checking was used to confirm the meaning of the dialogue during the review so that the themes that emerged were accurately reflected (Cresswell & Plano Clark, 2011).

## Results/Analysis

Six themes surfaced: confidence, empathy, communication, mentorship, self-knowledge, and cultural competency. The themes described were core elements of empowering providers towards advocacy and process improvement. Participants reported that the simulation experience resulted in an improved ability to respond to distressing situations and utilize soft skills. Table 2 represents participant feedback to support the themes.

**Table 2.** Participant feedback to support the six emerging themes

**Confidence**	• “The point I’m trying to make with this is that it ‘simulation’ helped me realize what I’m going into in another month and understanding that it’s certainly a quick pace.” (Debrief #2) • “I think the reason I was feeling like I couldn’t say anything was that my mind was just going through all the steps.” (Debrief #2) • “I’m kind of new to the evaluation process. I’ve learned everything in books, but I’ve never actually taken it into the clinic working with real patients. Getting the flow going.... To give them the best care we can in that one visit that we see them.” (Debrief #3) • “I just feel more secure going in to internships.” (Debrief #4) • “I was more confident in what questions I was going to ask.” (Debrief #4)
**Empathy**	• “I felt like I was kind of like deer in the headlights. I had that feeling and then I also, like when she started crying like I didn’t know what to do, and I would say I am a very empathetic person, but for some reason, I just stood there. I know I think it was the nerves and just not being in that situation before and, also the language barrier.” (Debrief #2) • “When the mother started crying, like I realized we’re all standing in front of her staring at her and she was crying, so I go over and just put my arm around her, and it was just maybe like a hug, but she wraps her arm around me and holds me for the rest of the time she is talking.”(Debrief #3) • “Just how in the simulation training the mother just broke down and cried. I mean, that happened with a patient here. She just started saying that she didn’t want to die. I mean, she definitely wasn’t going to, but like seeing the fear and tears.” (Debrief #4) • “When you’re asking a question, looking at the patient, not the translator, really looking into their eyes and whenever they’re answering.” (Debrief #4) • “So, from that (the simulated encounter), I learned that it’s OK to stop what you’re doing and to be with that person because they are human. You don’t have to just be ‘OK, I have to get my job done. I can’t talk to you. So, it was kind of cool to set down the paintbrush and just have this conversation and get to know her better and make that relationship.” (Debrief #4)
**Communication**	• “So, I wasn’t in the clinic today, so that part of the simulation was not the same for me. But, um, I will say there was a moment when I was at the house build, it was just me. I was painting the railings in one of the rooms and the translator and the mom came in while I was painting and wanted to talk with me, so I stopped what I was doing and had a really cool conversation with them, which kind of felt like in the simulation. We had a moment where the mom started crying and I totally froze and didn’t know what to do. So, from that I learned that it’s OK to stop what you’re doing and to be with that person because they are human. You don’t have to just be OK ‘I have to get my job done. I can’t talk to you’. So, it was kind of cool to set down the paintbrush and just have this conversation and get to know her better and make that relationship.” (Debrief #3) • “When you’re asking a question, looking at the patient, not the translator, really looking into their eyes and whenever they’re answering.” (Debrief #4)
**Mentorship**	• “I just have such a passion for what we do...I love watching others grow. It’s fun to stand back and watch others have a passion for this field and let them experience that.” (Debrief #2) • “And I felt like so poured into and just in different kinds of ways” ....being constantly encouraged (Debrief #4) • “The upper tris really helped me.” (Debrief #4) • “I didn’t feel like I was stepping in and then they pulled me because I was doing too much. I need to let you do more without stepping in.” (Debrief #2)
**Self-knowledge**	• “I really feel like God is asking me to work on openness because that’s something I struggle with... I don’t want to ask for help because I want to do it myself on a personal relationship...it sucks to be vulnerable.” (Debrief #3) • “You have to be so flexible and accommodating and adapt your treatment plan when you’re working with kids...constantly having to be flexible and adapt and meet them.” (Debrief #3) • “The personal growth is just even more eye-opening, but I always try not to judge a book by its cover. ...like just taking a step back and really make sure you don’t judge because you never know when someone had just an awful experience.” (Debrief #4)
**Cultural Competency**	• “Just having in the back of your head, that you realize that these people, they don’t have anything, just your presence there. They are so thankful for that.” (Debrief #2) • “Here I had to evaluate each patient and really figure out what would be the best thing to do for them given their circumstance.” (Debrief #4) • “I do feel prepared, but coming to a completely different...world from where we live... I was faced with stories and situations that just still blow your mind and break your heart and I cried..... I do feel prepared but also it opened your eyes and there’s just some things that you just can’t totally be prepared for.” (Debrief #4)

## Discussion

Overall, the qualitative data suggest that the pre-departure simulation experience helped our participants better respond to distressing situations during the week in Guatemala. One theme that appeared early in the debriefing sessions was confidence. Confidence boosting following healthcare simulation experiences is well-supported in the literature. Ohtake, Lazarus, Schillo, & Rosen (2013) noted significant increases in student physical therapist’s confidence levels following a simulated patient treatment session. Consistent with Ohtake et al.’s (2013) findings, study participants relayed a better understanding of work pace and possible clinical scenarios they would be facing in Guatemala following the predeparture simulated patient encounter. A more concrete understanding of the complex cultural and ethical challenges faced by patients in an under-resourced environment led to one participant’s expression of improved confidence level for the upcoming service-learning experience:

“The point I’m trying to make with this is that it ‘simulation’ helped me realize what I’m going into in another month and understanding that it’s certainly a quick pace.”

Psychosocial role-playing has been used to improve the soft skill responses of nursing students to emotionally challenging situations ( Liebrecht & Montenery, 2016). Liebrecht & Montenery (2016) concluded that “this increased awareness and insight allows students to respond in a more comforting, helpful, and professional manner in the clinical setting.” Responding empathetically to the patient in a moment of distress was a highly evident theme within the participant dialogue. Specifically, student participants initially demonstrated a complete lack of response to a culturally novel and emotionally charged situation within the simulated encounter. One student participant revealed the following in the debrief immediately following the simulation encounter:

“I felt like I was kind of like deer in the headlights. I had that feeling and then I also, like when she started crying like I didn’t know what to do, and I would say I am a very empathetic person, but for some reason, I just stood there. I know I think it was the nerves and just not being in that situation before and, also the language barrier.”

However, while onsite in Guatemala, student participants were able to respond in a highly empathetic manner when faced with a similar situation. One student participant commented during the week of service:

“When the mother started crying, like I realized we’re all standing in front of her staring at her and she was crying, so I go over and just put my arm around her, and it was just maybe like a hug, but she wraps her arm around me and holds me for the rest of the time she is talking.”

On the continuum of empathetic interaction in patient care, Lown (2014) found self-regulation of one’s emotional responses to be essential to providing appropriate and effective care. While the goal is to build highly empathetic, culturally compassionate providers, they must also be able to manage emotions, to act in the patient’s best interest. Student participants indicated a reconciliation of self-regulatory responses between the simulated encounter and the service-learning experience noting personal growth in this skill as a result. One student participant reported the following:

“From the sim lab, it was just really, real because (today in the clinic) there was a patient telling me about her history of abuse with her husband, and how he broke her ulna in a fight. And so, I had to really kind of control my empathy, you have to really keep it in check.”

Skillful communication is widely regarded as the mainstay of patient safety. Research has shown that simulation can be effective in enhancing communication skill development by promoting active listening ( Kim, Ko & Lee, 2012). All participants in our study consistently expressed concerns with the communication language barrier in anticipation of the upcoming travel. For many of the participants, the simulated encounter was their first experience working with an interpreter in the clinical environment. One clinician participant advised immediately following the simulated encounter:

“when you’re asking a question, looking at the patient, not the translator, really looking into their eyes and whenever they’re answering.”

Another student participant commented during the week in Guatemala:

“So, from that (the simulated encounter), I learned that it’s OK to stop what you’re doing and to be with that person because they are human. You don’t have to just be ‘OK, I have to get my job done. I can’t talk to you. So, it was kind of cool to set down the paintbrush and just have this conversation and get to know her better and make that relationship.”

Themes around mentorship emerged from both the student and clinician participants during the entire experience as the more experienced students and clinicians were mentoring the newer participants. For example, during the simulated encounter, one supervising clinician overstepped her role of simply providing student guidance. A decision was made from the control room to remove her from the simulation and allow the students to work through the scenario more independently. The clinician commented that she did not even realize she was directing the scene:

“I didn’t feel like I was stepping in and then they pulled me because I was doing too much. I need to let you do more without stepping in.”

Her commentary demonstrates growth in mentorship skills which carried forward into the onsite learning experiences in Guatemala.

Self-awareness of one’s strengths, weaknesses, and background within the context of a global perspective emerged as a predominant theme. Participants expressed awareness of their limited exposure to diverse cultural and socioeconomically deprived environments after the simulated experience and more so once on site in Guatemala. Self-knowledge is commonly thought to be the initial stage of personal growth, and participants expressed the service-learning experience, supported by the simulation experience, stirred significant maturation within them. One student participant commented on the final day in Guatemala:

“the personal growth is just even more eye-opening, but I always try not to judge a book by its cover. ..like just taking a step back and really make sure you don’t judge because you never know when someone had just an awful experience.”

One of the best ways to teach cultural competency is through service-learning ( Ekelman et al., 2003; Mu et al., 2016; Johnson & Howell, 2017). Logar et al., (2015) suggests that to maximize student preparation for international service-learning, simulation that uses scenarios that engages students with the ethical dilemmas and distinct cultural challenges of the host community should be implemented. Students strongly expressed that, while nothing can wholly prepare them for the intense cultural differences experienced while on a service-learning trip to a resource-limited country, the simulation activity contributed greatly to their feelings of preparedness. One student participant commented *:*

“I do feel prepared, but coming to a completely different..world from where we live.. I was faced with stories and situations that just still blow your mind and break your heart and I cried... I do feel prepared but also it opened your eyes and there’s just some things that you just can’t totally be prepared for.”

## Conclusion

Systematic approaches are needed to help new and experienced healthcare providers develop and maintain the soft skills required to deliver compassionate care ( Lown, 2014). Whether at home or abroad, moving from cultural awareness to cultural competency and further still to cultural compassion is a necessity for providing patient-centered, quality care. Utilizing simulation in preparation for service-learning experiences in resource-limited settings appears highly effective for enhancing soft skill development in rehabilitation clinicians; making great strides toward producing culturally compassionate providers.

## Take Home Messages

• Using simulation in pre-departure training for healthcare providers participating in international-service learning is best-practice.
• Simulation to enhance soft skills for international service-learning should include cultural scenarios that are specific to the host country.
• Simulation scenarios should be brief and focus on a few key components, such as understanding communication or active listening skills.

## Notes On Contributors

Suzanne Trotter is an assistant professor at The University of St. Augustine for Health Sciences. She earned a B.A. from Auburn University, and then completed a MPT degree from Emory University in 1995. In 2014 she received her Doctor of Science in Physical Therapy from Texas Tech University Health Science Center.

Sharon Dunnivan-Mitchell in an instructor at The University of St. Augustine for Health Sciences. She received a B.S. in Physical Therapy from The University of Texas Southwestern Medical Center in 1988 following completion of a marketing degree from The University of Texas in 1987. Her DPT was completed at The College of St. Scholastica in 2017.
