# Supplementary material for: Simulation Enhances Soft Skills Among Inter-Professionals Participating in an International Service-Learning Experience to a Resource-Limited Country
Source: MedEdPublish (2016). 2019 Feb 5;8:24. [Version 1] doi: 10.15694/mep.2019.000024.1 (PMC10712614; doi:10.15694/mep.2019.000024.1)
Supplement: Peer review history

# Reviewer report: Reviewer response for version 1

**DOI:** 10.21956/mep.19773.r29704

**Published:** 2019-02-06

**Authors:** Felix Silwimba

**Affiliations:** 1 University of Lusaka

This review has been migrated. The reviewer awarded 5 stars out of 5 This article has provided me with an insight in preparing students for international service learning. I work and practice in a low income country I would like to introduce domestic service learning. I have gotten an idea on simulation on preparing students for domestic service learning.

Reviewer Expertise:

NA

No decision status is available

# Reviewer report: Reviewer response for version 1

**DOI:** 10.21956/mep.19773.r29703

**Published:** 2019-02-05

**Authors:** Samal Nauhria

**Affiliations:** 1 Windsor University School of Medicine

This review has been migrated. The reviewer awarded 4 stars out of 5 Well presented and nicely written article on the latest trends. Medical education is transforming towards simulation and virtual techniques to incorporate competencies in healthcare providers. Above all, in resource-limited scenarios, technology definitely comes to the rescue.

Reviewer Expertise:

NA

No decision status is available

# Reviewer report: Reviewer response for version 1

**DOI:** 10.21956/mep.19773.r29705

**Published:** 2019-02-05

**Authors:** P Ravi Shankar

**Affiliations:** 1 American International Medical University

This review has been migrated. The reviewer awarded 4 stars out of 5 I read the article on how simulation can be used to enhance soft skills with great interest. The qualitative research methodology has been described in great detail. I am assuming that the debriefing sessions were individual ones and not conducted in a group. I would be interested in knowing more details about the simulation scenario. What measures were taken by the authors to ensure that the scenario replicates conditions that the students and clinicians would encounter in Guatemala? The tables add significantly to the information provided in the text. The authors can also briefly mention conditions specific to Guatemala which they are trying to recreate in their simulation. This well written article will be of interest to a broad group of medical educators.

Reviewer Expertise:

NA

No decision status is available

# Reviewer report: Reviewer response for version 1

**DOI:** 10.21956/mep.19773.r29702

**Published:** 2019-02-05

**Authors:** Trevor Gibbs

**Affiliations:** 1 AMEE

This review has been migrated. The reviewer awarded 4 stars out of 5 I enjoyed reading this paper. It was a very well constructed piece of work that covers a difficult area- the addition of cultural diversity and poorly resourced country made it more difficult but more realistic. I am not in favour of the use of the term soft skills because I believe it belittles the importance of those skills, but in the absence of better terminology , I felt that its use was appropriate for this paper and the authors did expand on what constitutes the term. As always in these qualitative studies, it becomes hard to structure responses, but with this paper I thought the authors' structuring very appropriate.Although I felt that the paper lacked an element of "where to now", I would hope that the authors continue to develop this important piece of work and conduct a longitudinal study of this first cohort of students to evaluate the longer term effects upon the students.

Reviewer Expertise:

NA

No decision status is available
